# Morphine promotes microglial activation by upregulating the EGFR/ERK signaling pathway

**DOI:** 10.1371/journal.pone.0256870

**Published:** 2021-09-14

**Authors:** Yaqiong Yang, Yu Sun, Rong Hu, Jia Yan, Ziheng Wang, Wenlong Li, Hong Jiang

**Affiliations:** Department of Anesthesiology, Shanghai Ninth People’s Hospital, Shanghai Jiao Tong University School of Medicine, Center for Specialty Strategy Research of Shanghai Jiao Tong University China Hospital Development Institute, Shanghai, PR China; Max Delbruck Centrum fur Molekulare Medizin Berlin Buch, GERMANY

## Abstract

Although they represent the cornerstone of analgesic therapy, opioids, such as morphine, are limited in efficacy by drug tolerance, hyperalgesia and other side effects. Activation of microglia and the consequent production of proinflammatory cytokines play a key pathogenic role in morphine tolerance, but the exact mechanisms are not well understood. This study aimed to investigate the regulatory mechanism of epidermal growth factor receptor (EGFR) on microglial activation induced by morphine in mouse microglial BV-2 cells. In this research, BV-2 cells were stimulated with morphine or pretreated with AG1478 (an inhibitor of EGFR). Expression levels of cluster of differentiation molecule 11b (CD11b), EGFR, and phospho-EGFR were detected by immunofluorescence staining. Cell signaling was assayed by Western blot. The migration ability of BV-2 cells was tested by Transwell assay. The production of interleukin-1beta (IL-1β) and tumor necrosis factor-alpha (TNF-α) in the cell supernatant was determined by ELISA. We observed that the expression of CD11b induced by morphine was increased in a dose- and time- dependent manner in BV-2 cells. Phosphorylation levels of EGFR and ERK1/2, migration of BV-2 cells, and production of IL-1β and TNFα were markedly enhanced by morphine treatment. The activation, migration, and production of proinflammatory cytokines in BV-2 cells were inhibited by blocking the EGFR signaling pathway with AG1478. The present study demonstrated that the EGFR/ERK signaling pathway may represent a novel pharmacological strategy to suppress morphine tolerance through attenuation of microglial activation.

## Introduction

Opioids, such as morphine, continue to be powerful analgesic drugs for managing chronic pain with a high rate of abuse potential [1]. Their analgesic efficacy is limited by drug tolerance, hyperalgesia and other side effects, which hinder the prolonged clinical use of opioid drugs [2]. Therapeutic strategies that can bolster the analgesia effects while reducing tolerance and side effects are urgently needed to improve patient quality of life.

Although the exact mechanisms of opioid tolerance are complex and not well understood, compelling evidence has recently shown that the activation of microglia and consequent production of proinflammatory cytokines play a key pathogenic role [3, 4]. Activated microglia undergo a dramatic morphological changes from quiescent to a macrophage-like accompanied by increased expression of cell surface markers, such as cluster of differentiation molecule 11b (CD11b), cluster of differentiation (CD14), major histocompatibility complex (MHC) molecules, chemokine receptors, and several other markers, producing large numbers of proinflammatory cytokines, including interleukin-1β (IL-1β), tumor necrosis factor-α (TNF-α), and IL-6, which could enhance the reactivity of dorsal horn neurons, induce central sensitization, and reduce the antinociceptive effect of morphine [5, 6]. Although the inhibition of microglial activation may represent a potential therapeutic strategy for improving the analgesic effect of morphine [7], the specific regulation mechanism is still unclear.

The epidermal growth factor receptor (EGFR) is a member of the ErbB family with four different receptor tyrosine kinases and has been successfully targeted for cancer therapy [8, 9]. EGFR can be activated by the Mu opioid receptors (MOR) [10, 11]. Inhibition of EGFR/MAPK signaling pathway attenuates microglial inflammatory response and secondary damage after spinal cord injury in rats [12]. EGFR may also be involved in pain and analgesia signaling in neuropathic pain rat models [13, 14]. However, none of these previous studies examined the role of EGFR in morphine induced microglial activation. Herein, we demonstrate that morphine induces activation of microglia in a dose- and time- dependent manner. Moreover, we found the impact of morphine on microglia was due to EGFR and its downstream ERK signaling pathway activation and that inhibition of EGFR suppresses the activation and migration of microglia and reduces the secretion of proinflammatory cytokines. These data suggest that EGFR may have potential as a novel target for the treatment of morphine tolerance.

## Materials and methods

### Cell culture

The mouse microglial BV-2 cell line was purchased from the Cell Bank of Type Culture Collection of the Chinese Academy of Sciences (Shanghai, China). Cells were cultured in Dulbecco’s modified Eagle’s medium (High Glucose, Gibco, China) with 10% heat-inactivated fetal bovine serum (FBS, Gibco, USA) and 1% penicillin-streptomycin (Sangon Biotech, Shanghai, China) at 37°C in a humidified atmosphere of 95% air and 5% CO_2_. When cells reached approximately 80% confluence, they were used for experiments.

### Cell treatments

Morphine hydrochloride (10 mg/mL) was obtained from Shenyang First Pharmaceutical Factory, Northeast Pharmaceutical Group Company (Shenyang, China) and diluted in phosphate-buffered saline (PBS, Gibco, China) to 1 mg/mL. Lipopolysaccharide (LPS) was purchased from Sigma-Aldrich (USA) and dissolved in PBS at a final concentration of 1 μg/ml according to previous studies [12, 15–19]. AG1478, a specific tyrosine kinase inhibitor of EGFR, was purchased from MedChemExpress (USA) and dissolved in 10% dimethyl sulfoxide (DMSO, Sigma-Aldrich, USA) at a final concentration of 2.5, 5, and 10 μM according to previous study [12]. Cells were pretreated with AG1478 for 30 min before treatment with other drugs.

### Immunofluorescence staining

Different groups of BV-2 cells were cultured at 2×10^5^ cells/cm^2^ on glass coverslips in 6-well culture plates. Coverslips were fixed with 4% paraformaldehyde for 15 min, blocked with 5% FBS for 30 min, and then incubated with CD11b antibody (1:200, ab8878, Abcam, UK), anti-EGFR (1:250, ab52894, Abcam, UK) or anti-phospho-EGFR (Tyr1068) (1:500, 3777, Cell Signaling Technology, USA) at -4°C overnight. After being washed, cells were incubated with the corresponding fluorescent-conjugated IgG secondary antibody (Proteintech Group, PTG, USA) at 37°C for 1 h and then counterstained with Hoechst (Beyotime Biotechnology, Shanghai, China) at room temperature in the dark for 15 min. Images were acquired under a fluorescence microscope (OLYMPUS IX71, Tokyo, Japan) and were analyzed using ImageJ software.

### Western blot analysis

Different groups of BV-2 cells were seeded and incubated in 6-well plates in DMEM with 10% FBS. For CD11b expression experiments, cells were incubated with 0, 25, 50, 100, and 200 μM morphine for 24 h or 200 μm morphine for 0, 2, 4, 6 and 8 h. For EGFR inhibition experiments, cells were incubated with DMSO, AG1478 (10 μM), morphine (200 μM), or AG1478 (10 μM) + morphine (200 μM) for 24 h. After treatments, total proteins were extracted after lysis in RIPA lysis buffer (Beyotime, China). Lysates were centrifuged at 12,000 g for 10 min and supernatants were collected for protein concentration assay, performed using a BCA kit (Pierce, Rockford, IL). Proteins (20 μg) were separated on 8% SDS-PAGE gels and transferred to polyvinylidene fluoride membranes (Millipore Billerica, MA). The membranes were blocked with 5% skim milk and incubated overnight at 4°C with primary antibodies: anti-CD11b (1:1000, A1581, ABclonal, China), anti-EGFR (1:1000, ab52894, Abcam, UK), anti-phospho-EGFR (Tyr1068) (1:1000, 3777, Cell Signaling Technology, USA), anti-ERK1/2 (1:5000, ab184699, Abcam, UK), anti-phospho-ERK1/2 (1:2000, 4370, Cell Signaling Technology, USA), anti-TLR4 (1:1000, AF7017, Affinity Biosciences, China) and anti-GAPDH (1:2000, AF7021, Affinity Biosciences, China). Next, membranes were incubated with horseradish peroxidase-conjugated goat anti-rabbit IgG secondary antibodies (Jackson ImmunoResearch, USA) for 1 h at room temperature with subsequent exposure of membranes to enhanced chemiluminescence reagents (Millipore Billerica, MA).

### Migration assay

Transwell® migration assays were performed in 24-well Transwell® polycarbonate membrane inserts with 8.0-mm pore size (diameter 6.5 mm, Costar, Corning Incorporated, USA). BV-2 cells (100 μl, 1x10^5^ cells/well) were loaded into the upper chamber and allowed to adhere to the polycarbonate filters for 10 min at 37°C in a humidified atmosphere of 95% air and 5% CO_2_. According to the grouping, 200 μM morphine was added to the upper chamber in the morphine group and morphine + AG1478 group, and 10 μM AG1478 was added to the upper and lower chambers in both the AG1478 group and morphine + AG1478 group. Equal amount of DMSO was added in the control group. Cells were cultured at 37°C for 48 h. Then, migrated cells were fixed with 4% paraformaldehyde, prechilled on ice for 30 min, and stained with 0.1% crystal violet for 10 min. Six fields of view were randomly selected under a microscope (OLYMPUS IX71, Tokyo, Japan), and the number of migrated cells was counted at a 20 μm scale bar.

### Enzyme-linked immunosorbent assay (ELISA)

BV-2 cells (1×10^4^ cells/well) were cultured in 96-well plates overnight. Cells were treated with PBS, DMSO, LPS (1 μg/mL), morphine (200 μM), LPS (1 μg/mL) + morphine (200 μM), morphine (200 μM) + AG1478 (10 μM), and LPS (1 μg/mL) + morphine (200 μM) + AG1478 (10 μM) for 3, 6, 12, and 24 h, respectively. Levels of IL-1β and TNF-α in cell supernatants of different groups were measured using a Mouse Interleukin-1β ELISA Kit (LianShuo Biological, Shanghai, China) and a Mouse TNF-α ELISA Kit (LianShuo Biological, Shanghai, China), according to the manufacturer’s protocols. The absorbance of each well was measured at 450 nm using a microplate leader.

### Statistical analysis

All experiments were completed at least three times and data are presented as the mean ± standard error of the mean (SEM). Data were analyzed using analysis of variance (ANOVA) and the least significant differences method for multiple comparisons. *p* < 0.05 was considered statistically significant.

## Results

### Morphine induces microglial activation and enhances CD11b expression

**Fig 1:**
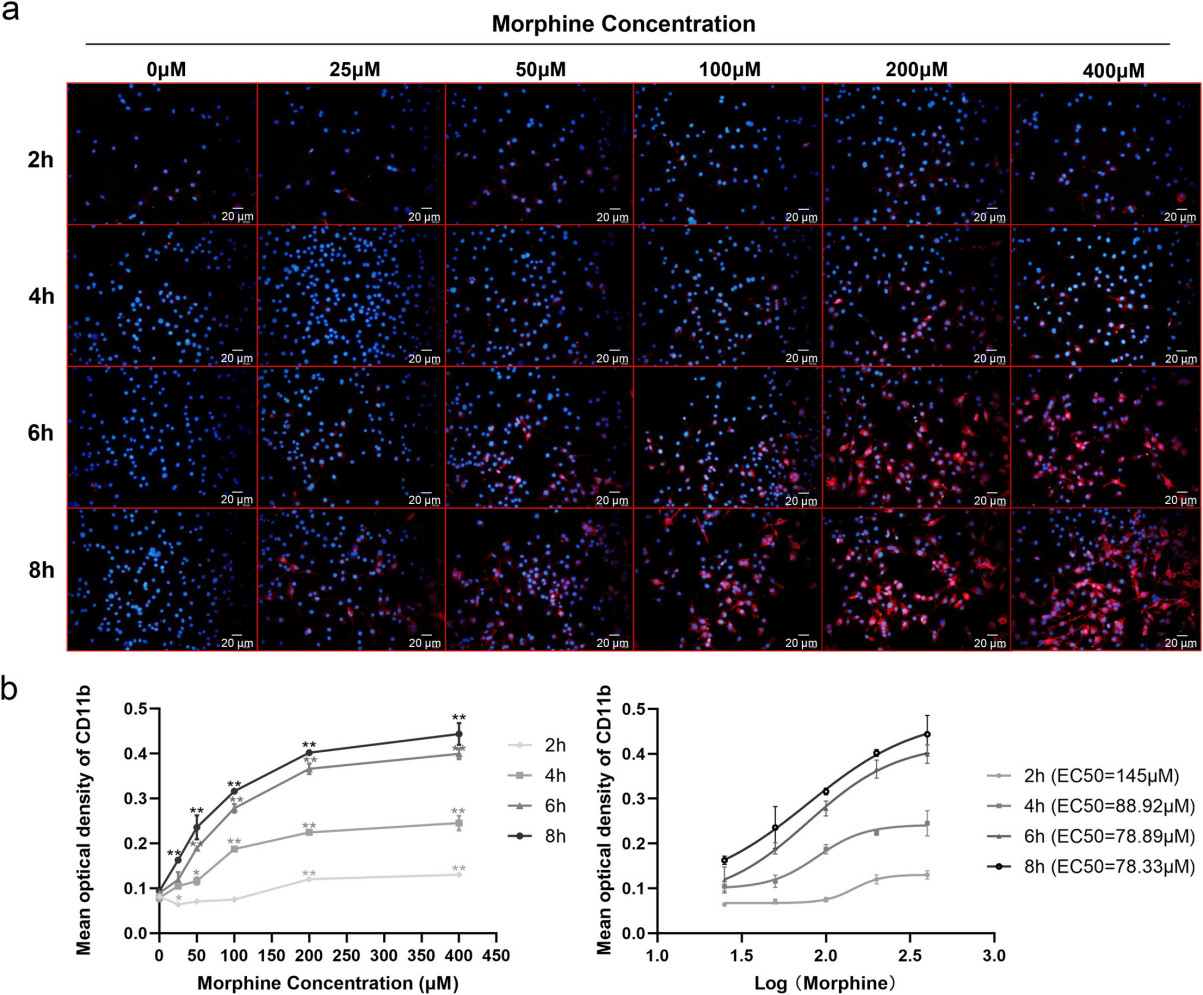
Morphine upregulates CD11b in a dose- and time-dependent manner in microglial BV-2 cells. BV-2 cells were treated with 0, 25, 50, 100, 200, and 400 μM morphine for 2, 4, 6, and 8 h and tested by immunocytochemistry staining. (a) Time- and dose-response effect of morphine on the expression of CD11b. Scale bar: 20 μm. (b) The time- and dose-response curve of BV-2 cells activated by morphine. Data were fit to a sigmoidal dose-response curve and EC50 values calculated using GraphPad Prism software. The mean optical densities of six randomly selected fields of cells from each group were analyzed by ImageJ software for quantitative analysis. Data are expressed as the means ± SEMs. All data were analyzed using ANOVA. * *p* < 0.05 and ** *p* < 0.01 were considered to be statistically significant compared to the control groups.

**Fig 2:**
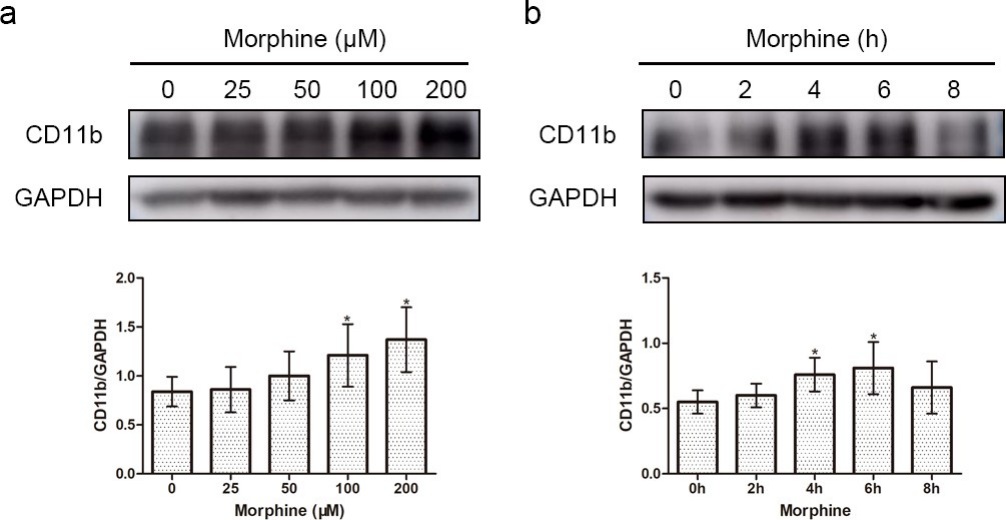
Dose- and time-dependent changes in the expression of CD11b proteins with morphine treatment were analyzed by Western blotting. BV-2 cells were treated with 0, 25, 50, 100, and 200 μM morphine for 24 h (a) or with 200 μm morphine for 0, 2, 4, 6 and 8 h (b). GAPDH was used as a loading control. Data are expressed as the means ± SEMs. All data were analyzed using ANOVA. * *p* < 0.05 compared to the control group.

To determine the effect of morphine on induced microglial activation, we used the immortalized murine microglial BV-2 cell line, which was derived from primary mouse microglial cells and has a similar responsive pattern to that of primary microglia after stimulation with LPS [20]. BV-2 cells were treated with different doses of morphine (0, 25, 50, 100, 200, and 400 μM) for 2, 4, 6, and 8 h, followed by immunofluorescence and Western blot analysis using CD11b, which is a microglial activation marker. From the results, we observed dose- and time- dependent effects of morphine on the expression of CD11b (Figs 1 and 2). EC50 values for each time points of morphine action were: 2 h, 145 μM; 4 h, 88.92 μM; 6 h, 78.89 μM; 8 h, 78.33μM (Fig 1B). Morphine, when used at a concentration of 200 μM for 6 h, caused microglia cells to exhibit an enlarged morphology and displayed intense CD11b immunoreactivity (Figs 1 and 2B). Previous studies have shown that morphine (200 μM, 6 h) significantly induced the activation of BV-2 cells [7, 21], which was consistent with our results. However, when BV-2 cells were treated with 200 μM morphine for 8 h, protein expression of CD11b was decreased (Fig 2B). Therefore, we chose a morphine concentration of 200 μM and a time of 6 h for subsequent experimental conditions.

### EGFR inhibition blocks the activation of microglia induced by morphine

**Fig 3:**
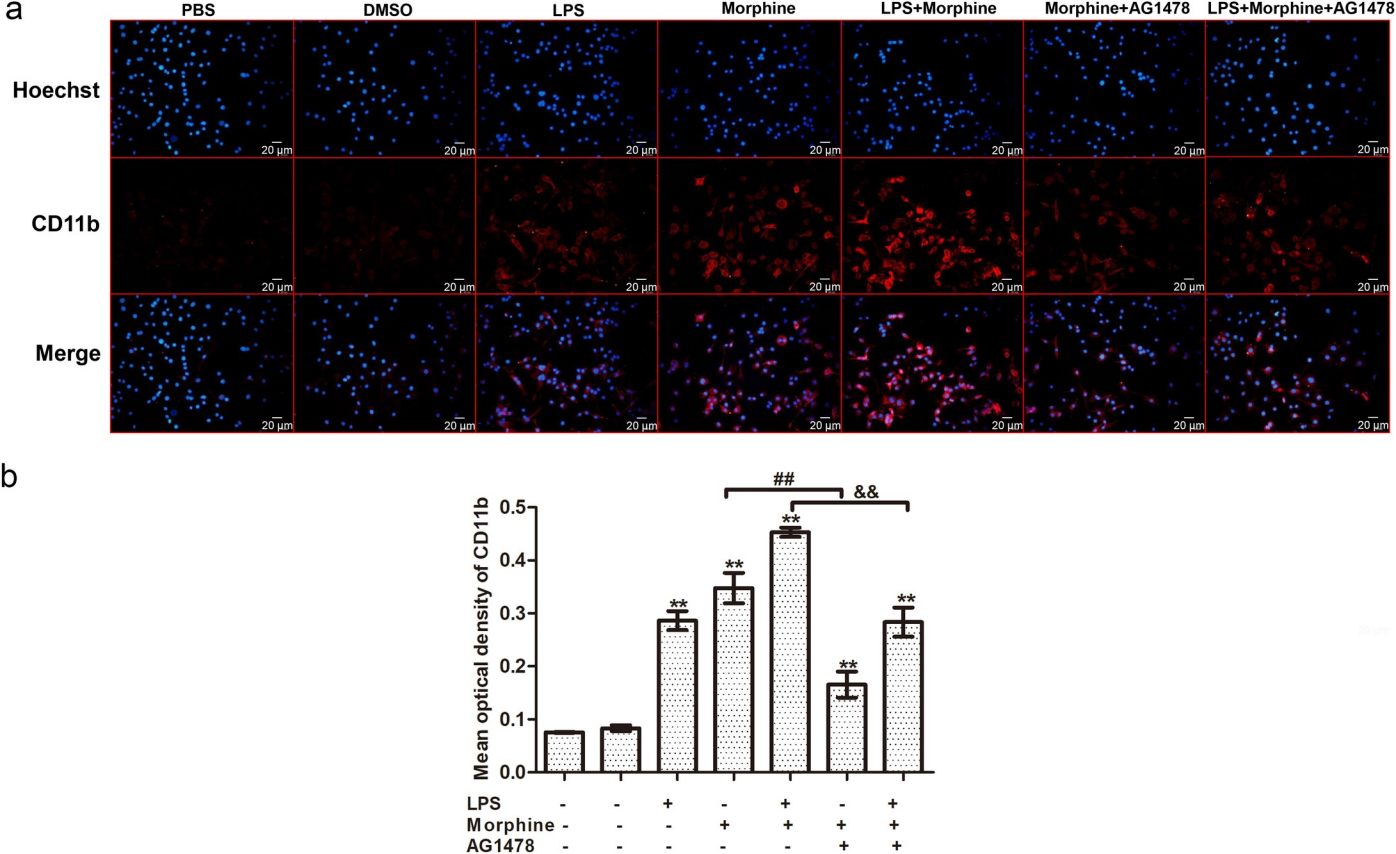
The effect of morphine and LPS on microglial activation by immunocytochemical analysis. (a) BV-2 cells were treated with PBS, DMSO, LPS (1 μg/mL), morphine (200 μM), LPS (1 μg/mL) + morphine (200 μM), morphine (200 μM) + AG1478 (10 μM), and LPS (1 μg/mL) + morphine (200 μM) + AG1478 (10 μM) for 6 h. Cells were stained for the activation marker CD11b. Scale bar: 20 μm. (b) The mean optical densities of six randomly selected fields of cells from each group were analyzed by ImageJ software for quantitative analysis. Data are expressed as the means ± SEMs. All data were analyzed using ANOVA. * *p* < 0.05 and ** *p* < 0.01 compared to the control groups. ^#^ *p* < 0.05 and ^##^ *p* < 0.01 morphine vs. morphine + AG1478. ^&^ *p* < 0.05 and ^&&^ *p* < 0.01 LPS + morphine vs. LPS + morphine + AG1478.

To examine the activation of microglia by treatment with morphine or LPS, we detected the expression of CD11b in BV-2 cells by immunofluorescence. LPS has been the most extensively used microglia activator to induce inflammatory response [22]. Morphine also increased LPS-induced cytokine production in cultivated microglia [23]. Previous study has shown that EGFR inhibitors depressed inflammation after LPS stimulation by regulating the activation of EGFR/MAPK cascade in microglia [16], which may be a new neuroprotective mechanism after EGFR blockade. For these reasons, we used LPS as a positive control to study the effects of EGFR inhibitor on LPS- and morphine- activated microglia. Ours results demonstrated that treatment with morphine and LPS both increased the expression of CD11b on microglial membranes, and induced BV-2 cells activation and macrophage-like morphologically changes (Fig 3). We also found that the combination of morphine and LPS could further enhance the activation of microglia (Fig 3). Pretreatment with the EGFR inhibitor AG1478 abolished microglial activation in both the morphine and LPS + morphine groups [Fig 3, morphine (0.35 ± 0.03) vs. morphine + AG1478 (0.17 ± 0.02), *p*<0.01; LPS + morphine (0.45 ± 0.01) vs. LPS + morphine + AG1478 (0.28 ± 0.03), *p*<0.01]. AG1478 alone had no significant effect on the expression of CD11b at different concentrations (S1 Fig).

### Morphine enhances expression of EGFR in microglia

**Fig 4:**
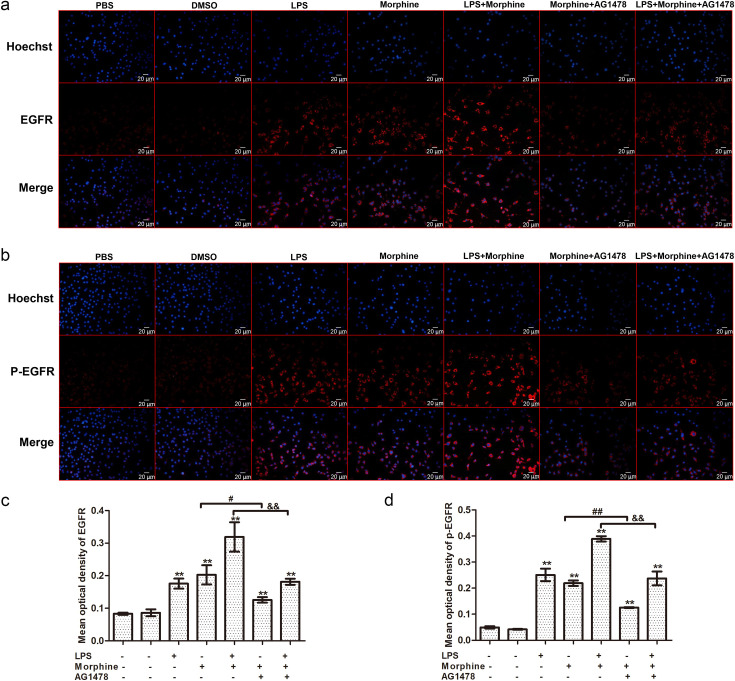
The effect of morphine and LPS on expression of EGFR and p-EGFR. The expression of EGFR (a) and p-EGFR (b) in BV-2 cells was detected by immunocytochemical analysis. Scale bar: 20 μm. (c), (d) The mean optical densities of six randomly selected fields of cells from each group were analyzed by ImageJ software for quantitative analysis. Data are expressed as the means ± SEMs. All data were analyzed using ANOVA. * *p* < 0.05 and ** *p* < 0.01 compared to the control groups. ^#^ *p* < 0.05 and ^##^ *p* < 0.01 morphine vs. morphine + AG1478. ^&^ *p* < 0.05 and ^&&^ *p* < 0.01 LPS + morphine vs. LPS + morphine + AG1478.

In this series of experiments, we sought to determine whether morphine directly induces EGFR expression in microglia. Accompanied by microglial activation, treatment with morphine (200 μM) or LPS (1 μg/mL) for 6 h significantly increased EGFR and p-EGFR expression, which was further increased by combined treatment of morphine and LPS (Fig 4, compared to the control group, *p*<0.01). Morphine-induced EGFR expression was reduced in response to pretreatment with AG1478 [Fig 4C, morphine (0.20 ± 0.03) vs. morphine + AG1478 (0.13 ± 0.01), *p*<0.05; LPS + morphine (0.32 ± 0.05) vs. LPS + morphine + AG1478 (0.18 ± 0.01), *p*<0.01]. Expression of p-EGFR was also inhibited by AG1478 [Fig 4D, morphine (0.22 ± 0.01) vs. morphine + AG1478 (0.13 ± 0.01), *p*<0.01; LPS + morphine (0.39 ± 0.01) vs. LPS + morphine + AG1478 (0.24 ± 0.03), *p*<0.01].

### Morphine induces microglial activation through the EGFR/ERK pathway

**Fig 5:**
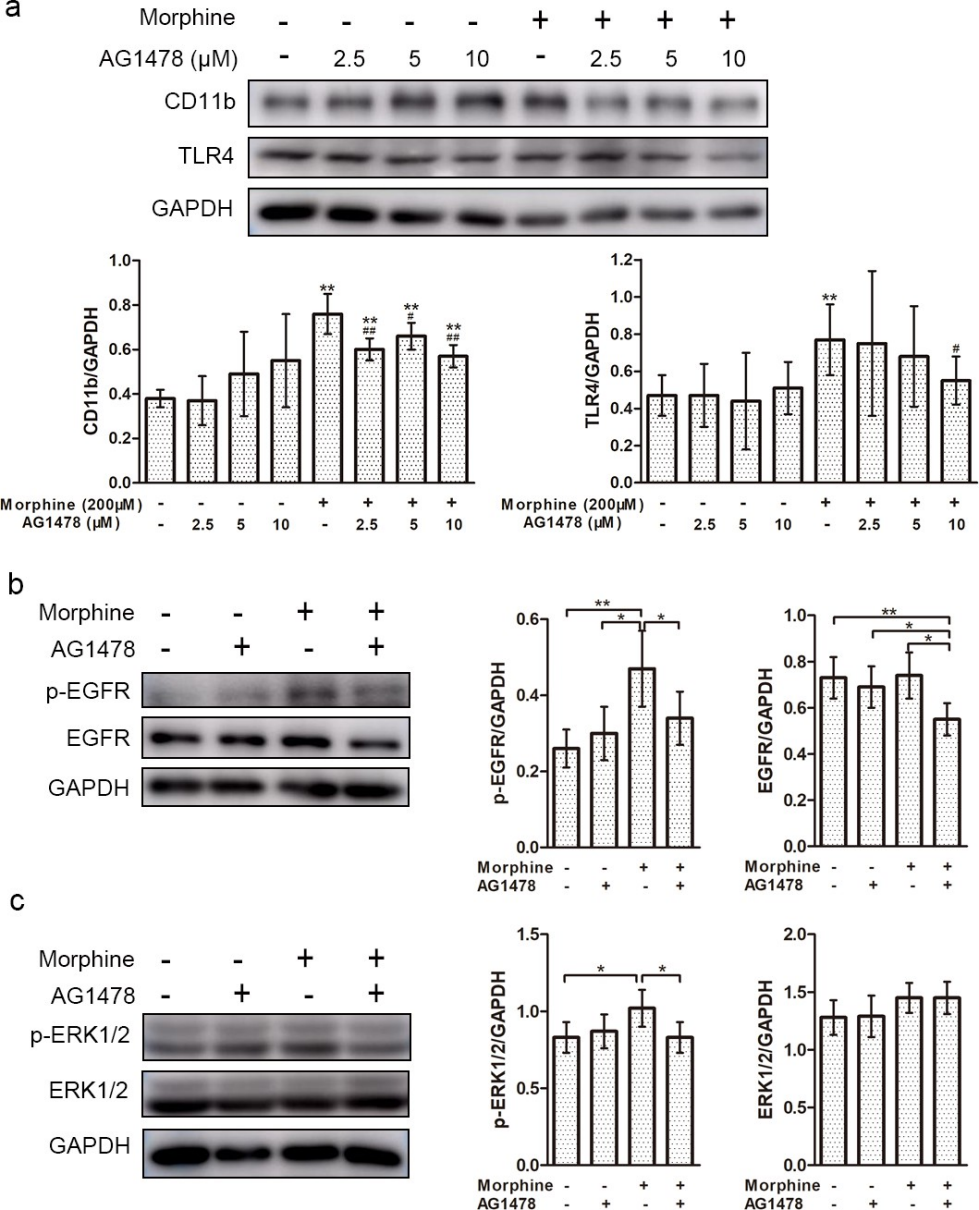
The effect of EGFR inhibition on morphine-induced protein expression. BV-2 cells were incubated with DMSO, AG1478 (2.5, 5, and 10 μM), morphine (200 μM), and AG1478 (2.5, 5, and 10 μM) + morphine (200 μM) for 24 h. After treatment, cell lysates were examined by Western blot for expression levels of CD11b (a), EGFR and p-EGFR (b) and ERK1/2 and p-ERK1/2 (c). GAPDH was used as a loading control. Data are expressed as the means ± SEMs. All data were analyzed using ANOVA. * *p* < 0.05 and ** *p* < 0.01 were considered to be statistically significant.

To further explore whether morphine upregulation of CD11b expression occurs through activation of the EGFR signaling pathway, BV-2 cells were pretreated with AG1478 for 30 min prior to treatment with morphine. Western blotting results revealed that protein expression of CD11b was significantly increased when cells were treated with morphine and markedly inhibited by AG1478 (Fig 5A). Morphine increased phosphorylation levels of EGFR but had no significant effect on total levels of EGFR. Treatment with AG1478 reversed the increase in EGFR levels induced by morphine (Fig 5B). We also found that expression of p-ERK1/2 was clearly enhanced by morphine treatment and decreased by AG1478. However, total levels of ERK1/2 did not obviously change in response to treatment (Fig 5C). These results suggest that morphine may activate microglia by regulating the EGFR/ERK pathway. Studies indicated that morphine could bind to Toll-like receptor 4 (TLR4) and induced the initiation of innate immune signaling cascade and the production of proinflammatory factors [23], which activated neuroinflammation in a manner parallel to endotoxin. Therefore, we also examined the effect of AG1478 on morphine induced TLR4 expression. The result found that AG1478 downregulated the expression of TLR4 induced by morphine (Fig 5A), which indicated that AG1478 might inhibit the activation of microglia by blocking innate immune signaling pathway.

### EGFR inhibition reduces microglial migration induced by morphine

**Fig 6:**
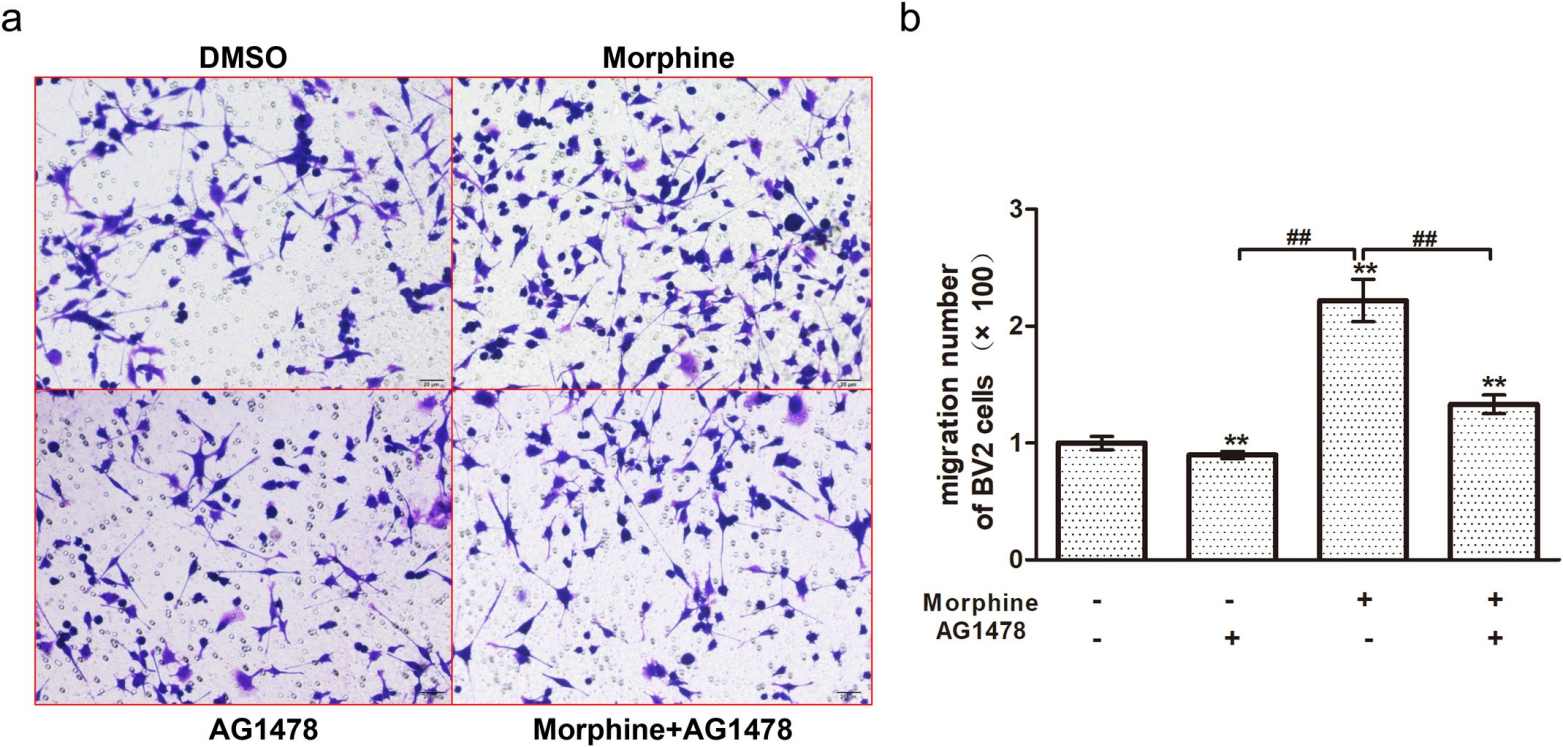
The effect of EGFR inhibition on migration of BV-2 cells induced by morphine. (a) BV-2 cells were treated with morphine or AG1478 for 48 h, and the number of migrated cells was evaluated by the Transwell assay. Scale bar: 20 μm. (b) Migration of BV-2 cells in six randomly selected fields from each group was quantified. Data are expressed as the means ± SEMs. All data were analyzed using ANOVA. * *p* < 0.05 and ** *p* < 0.01 compared to DMSO groups. ^##^ *p* < 0.01 morphine vs. morphine + AG1478.

To confirm the effect of EGFR inhibition on the migration of microglia induced by morphine, the migration ability of cells was evaluated by Transwell assay. The results showed that after treatment with morphine, the number of migrated microglia was obviously increased [morphine (222 ± 18) vs. DMSO (100 ± 6), *p* <0.01], and AG1478 significantly inhibited microglial invasion [morphine (222 ± 18) vs. morphine+AG1478 (133 ± 8), *p* < 0.01] (Fig 6).

### AG1478 inhibits morphine- and LPS- induced IL-1β and TNF-α production in BV-2 microglial cells

**Fig 7:**
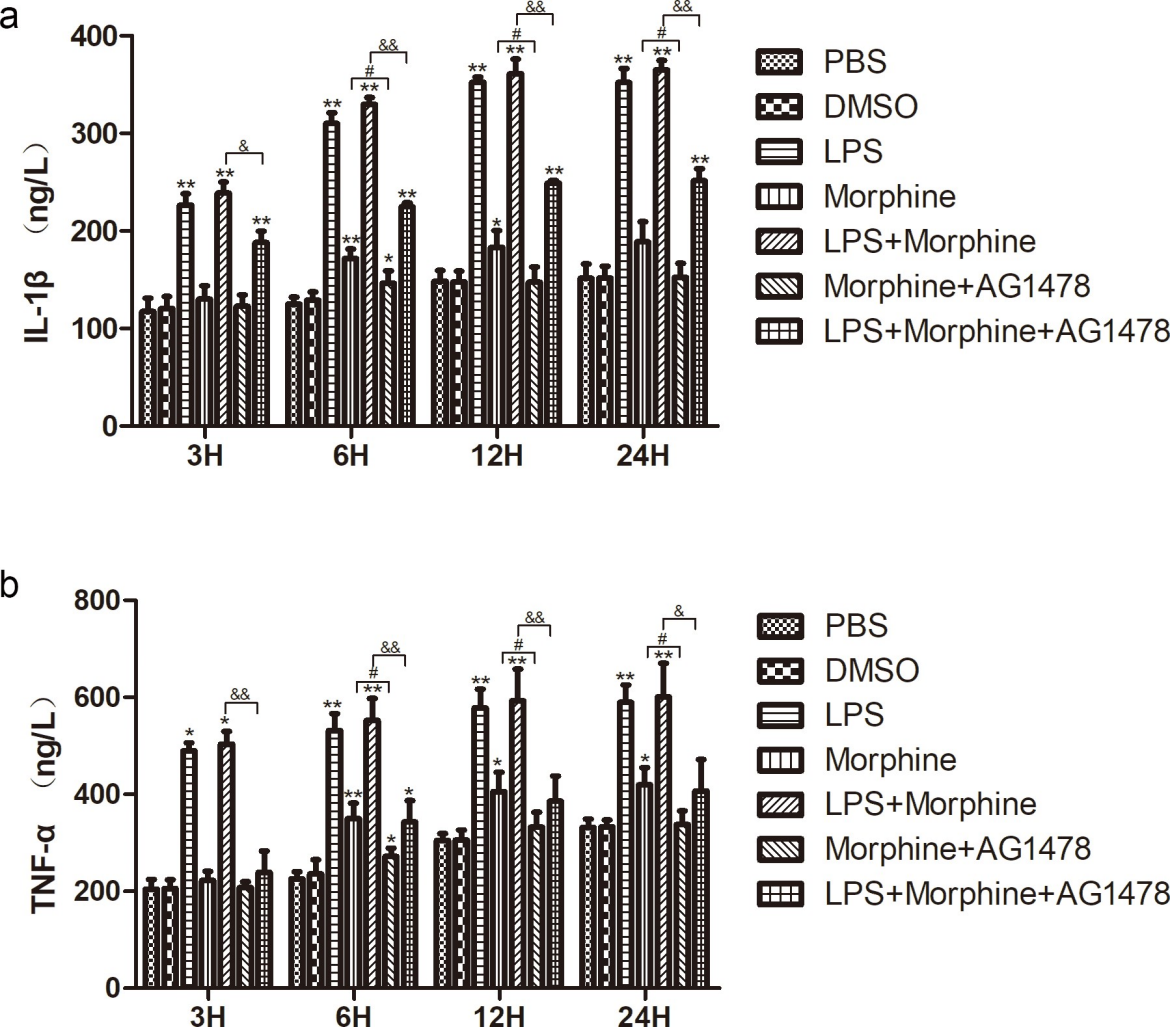
**The effect of EGFR inhibition on morphine- and LPS- induced IL-1β (a) and TNF-α (b) production in BV-2 microglial cells.** The supernatants of BV-2 cells were collected after centrifugation and measured by ELISA. The absorbance of each well was measured at 450 nm using a microplate leader. Data are presented as the means ± SEMs of three independent experiments. * *p* < 0.05 and ** *p* < 0.01 compared to PBS groups. ^#^ *p* < 0.05 and ^##^ *p* < 0.01 morphine vs. morphine + AG1478. ^&^ *p* < 0.05 and ^&&^ *p* < 0.01 LPS + morphine vs. LPS + morphine + AG1478.

To further explore whether morphine- and LPS- mediated microglial activation is accompanied by an inflammatory response in BV-2 cells, we investigated the secretion of the key proinflammatory cytokines, IL-1β and TNF-α by ELISA analysis in cellular supernatants. Our results showed that LPS time-dependently increased the production of the inflammatory mediators IL-1β and TNF-α by BV-2 cells. Morphine increased the production of IL-1β at 6 h and 12 h compared to the PBS group, but there was no significant difference at 3 h or 24 h (Fig 7). Levels of TNF-α induced by morphine increased gradually from 6 h after administration. We also found that treatment with AG1478 markedly attenuated IL-1β and TNF-α production induced by morphine and LPS + morphine (Fig 7).

## Discussion

Although more and more pieces of literature show that spinal microglia play an important role in the pathogenesis of morphine tolerance [24, 25], but the exact regulatory mechanism is not clear. The study presented here assessed the signaling mechanisms by which morphine modulates microglial activation. A previous study suggested that morphine at concentrations >1 μM acts as a microglial chemoattractant in a rodent model of neuropathic pain [26]. Our results indicated that morphine potently enhanced expression of the microglial marker CD11b in a dose- and time-dependent manner, consistent with a previous study. It has been shown that there is remarkable resemblance between neuropathic pain and morphine tolerance. The activation of neuroimmunity was more obvious in rats with nerve injury and morphine than with injury alone, indicating that opioids and neuropathy may have cooperative effects on glial activation and cytokines [27]. In this study, we used morphine and LPS alone or in combination to treat BV-2 microglial cell. We found that morphine improved microglial reactivity, induced migration, and increased the expression of CD11b. LPS also upregulated the expression of CD11b, which was further increased by combined treatment with morphine. These results suggest that the activation process of microglia induced by morphine may have the same mechanism as that of inflammatory activation induced by LPS.

EGFR, a well-known receptor, has been attracting much attention due to its potency in mediating microglial activation [28]. Binding to EGFR ligands, such as epidermal growth factor (EGF) and tumor necrosis factor α (TNF-α), causes microglial activation by transactivation of mitogen-activated protein kinase (MAPK) and other downstream signaling pathways [29, 30]. Inhibition of the EGFR/MAPK signaling pathway has been proven to suppress the microglial inflammatory response and associated secondary injury in rats after spinal cord injury [12], and rapid phosphorylation of EGFR is attributed to LPS-triggered intracellular calcium mobilization and migration of microglia [16]. However, the EGFR signaling pathway is essential for microglial activation and cytokine production, making it a potential therapeutic target for the treatment of inflammatory diseases, but the regulatory role of EGFR in morphine-induced microglial activation remains unclear. LPS, a natural TLR4 ligand, has been the most widely used microglia activator to induce inflammatory response [22]. Study has shown that LPS-induced microglia activation is accompanied by EGFR transactivation [12], but there is no evidence that LPS is an effective ligand for EGFR [16]. In this study, we found that morphine and LPS both remarkably upregulated levels of EGFR and p-EGFR in BV-2 cells and were further increased by combined treatment of morphine and LPS. Morphine- and LPS- induced EGFR and p-EGFR overexpression were reduced by pretreatment with AG1478, an inhibitor of EGFR (Fig 4). Furthermore, we found that protein phosphorylation levels of EGFR and ERK1/2 were both increased by morphine and decreased by AG1478 (Fig 5). The increased expression of CD11b induced by morphine was also inhibited by pretreatment with AG1478 (Figs 3 and 5A). These results indicate that EGFR pathway plays an important role in the process of microglia activation induced by morphine and LPS. Extensive studies have shown that opioids cause activation of signaling by the innate immune receptor TLR4, leading to proinflammatory glial activation [23, 31–33]. Blockade of TLR4 inhibited the microglia activation and attenuated morphine tolerance [34]. In this study, AG1478 also inhibited the expression of TLR4 induced by morphine, suggesting that EGFR inhibitors depress microglia activation through regulating innate immune signaling pathway.

Morphine-induced activation of microglia may lead to elevated local concentrations of proinflammatory cytokines and chemokines [2, 35, 36], hindering the efficiency of morphine analgesia [37]. Proinflammatory cytokines secreted by continuously activated microglia include IL-1β and TNF-α [38]. IL-1β is one of the most crucial members of IL-1 family and an angiogenic agent involved in the mechanism of neuropathic pain. In addition, the recent research has shown that IL-1β is the first cytokine involved in the development of morphine tolerance [39]. Blockade of IL-1 or IL-1β signaling can obviously reduce the development of morphine tolerance [40, 41]. TNF-α is an effective proinflammatory cytokine that can be expressed by glial cells [42]. Inhibition of TNF-α signaling suppresses the development of morphine tolerance in animal experiments [43–45]. Interestingly, a previous study found that morphine activates Toll-like receptor-4 (TLR-4) to elevate the production of proinflammatory cytokines in BV-2 cells in a way similar to LPS [46]. In the present study, we demonstrated that morphine treatment notably increased IL-1β and TNF-α produced by BV-2 cells, which is parallel to the previous reports [7, 47]. Morphine and LPS promoted activated microglia to produce increased proinflammatory factors, which was abolished by AG1478 pretreatment. Thus, our data suggest that EGFR inhibitors might suppress morphine-induced microglial activation primarily by inhibiting the production of proinflammatory cytokines.

In summary, we report that the EGFR signaling pathway is essential for microglial activation, metastasis and cytokine production, making it a potential therapeutic target for the treatment of morphine-induced drug tolerance. Due to the pleiotropic cellular function of the receptor family, the mechanism of EGFR mediated or promoted the occurrence and maintenance of neuropathic pain in a variety of mechanisms [48]. In our previous experiments, we found that cetuximab, an anti-EGFR monoclonal antibody, combined with celecoxib, a highly selective nonsteroidal anti-inflammatory agent, may help relieve cancer pain through reduced tumor-derived mediators *in vitro* [49]. In addition, in our recent study, we established cancer induced bone pain rat model with morphine tolerance and found that EGFR and its downstream ERK pathway were significantly activated in spinal cord after morphine administration [50]. EGFR is one of the most important targets of antitumor therapy, and EGFR antagonists may eventually play an indispensable clinical role not only in the treatment of malignant tumors but also in the treatment of pain, especially cancer pain, which is resistant to opioid treatment.

## Supporting information

S1 Fig The expression of CD11b in BV2 microglia treated with AG1478. (a) BV-2 cells were treated with DMSO, AG1478 (2.5, 5, and 10 μM), morphine (200 μM), and AG1478 (2.5, 5, and 10 μM) + morphine (200 μM) for 6 h and tested by immunocytochemistry staining (red fuorescence; × 200 magnifcation). (b) The mean optical densities of six randomly selected fields of cells from each group were analyzed by ImageJ software for quantitative analysis. Data are expressed as the means ± SEMs. All data were analyzed using ANOVA. * *p* < 0.05 and ** *p* < 0.01 compared to the DMSO group. ^#^ *p* < 0.05 and ^##^ *p* < 0.01 compared to morphine group.
(TIF)

S1 Raw images (PDF)
