# Supplementary figures and images for: Morphine promotes microglial activation by upregulating the EGFR/ERK signaling pathway
Source: PLoS One. 2021 Sep 14;16(9):e0256870. doi: 10.1371/journal.pone.0256870 (PMC8439491; doi:10.1371/journal.pone.0256870)

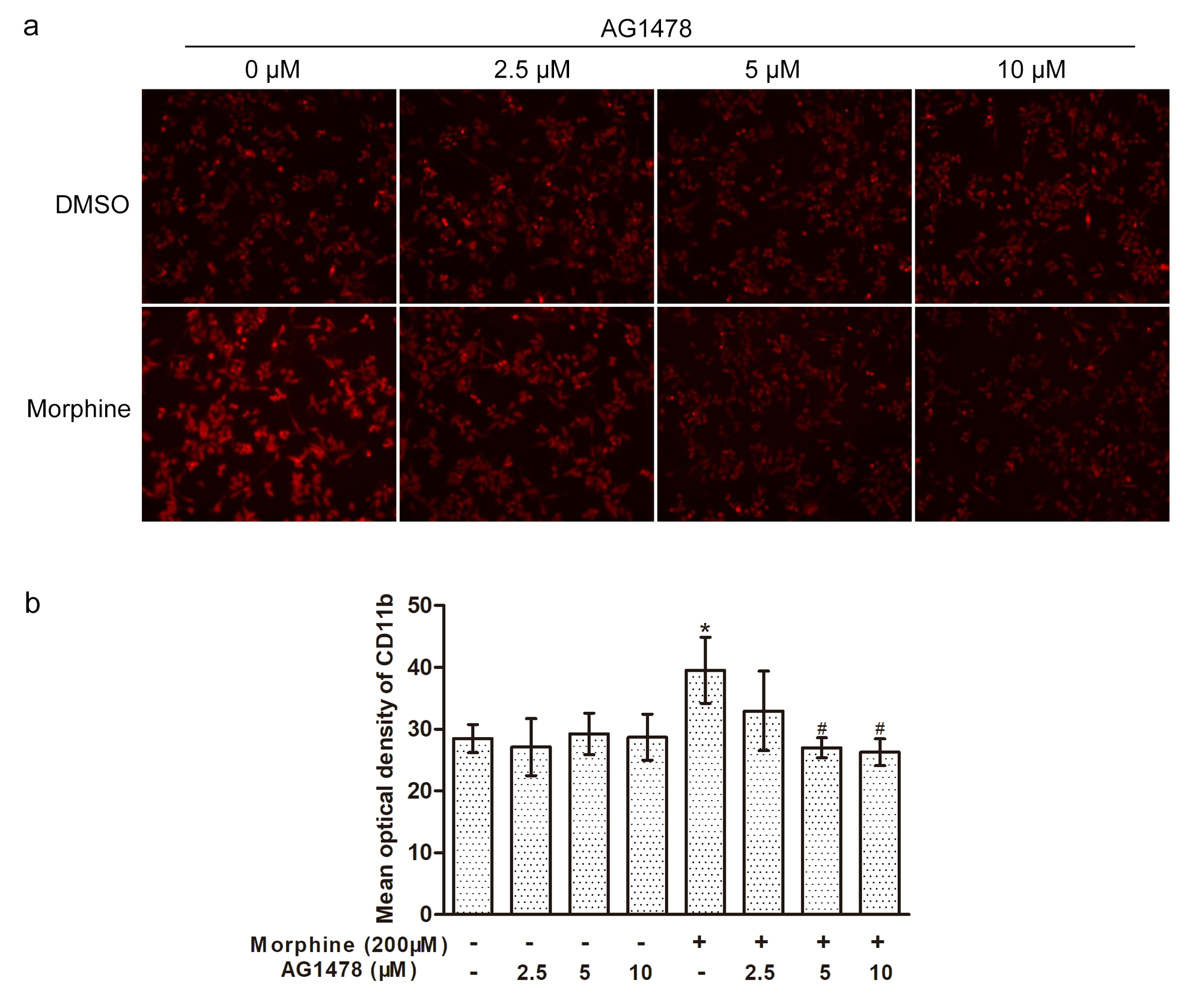

Supplement: S1 Fig — (a) BV-2 cells were treated with DMSO, AG1478 (2.5, 5, and 10 μM), morphine (200 μM), and AG1478 (2.5, 5, and 10 μM) + morphine (200 μM) for 6 h and tested by immunocytochemistry staining (red fuorescence; × 200 magnifcation). (b) The mean optical densities of six randomly selected fields of cells from each group were analyzed by ImageJ software for quantitative analysis. Data are expressed as the means ± SEMs. All data were analyzed using ANOVA. * p < 0.05 and ** p < 0.01 compared to the DMSO group. # p < 0.05 and ## p < 0.01 compared to morphine group. (TIF) [file pone.0256870.s001.tif]
