# Supplementary material for: Morphine promotes microglial activation by upregulating the EGFR/ERK signaling pathway
Source: PLoS One. 2021 Sep 14;16(9):e0256870. doi: 10.1371/journal.pone.0256870 (PMC8439491; doi:10.1371/journal.pone.0256870)

Figure 2a

Figure 2b

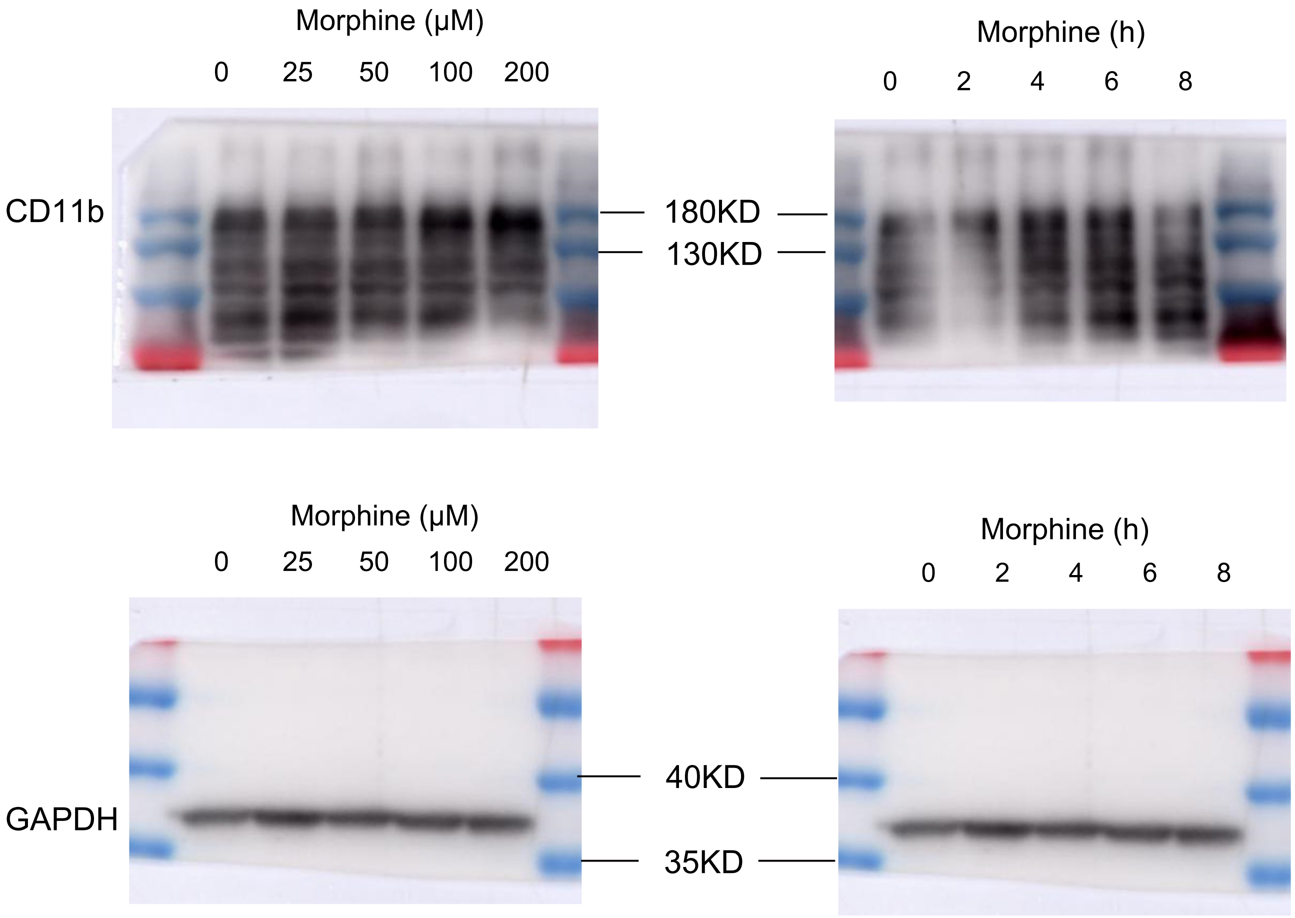

Figure 5a

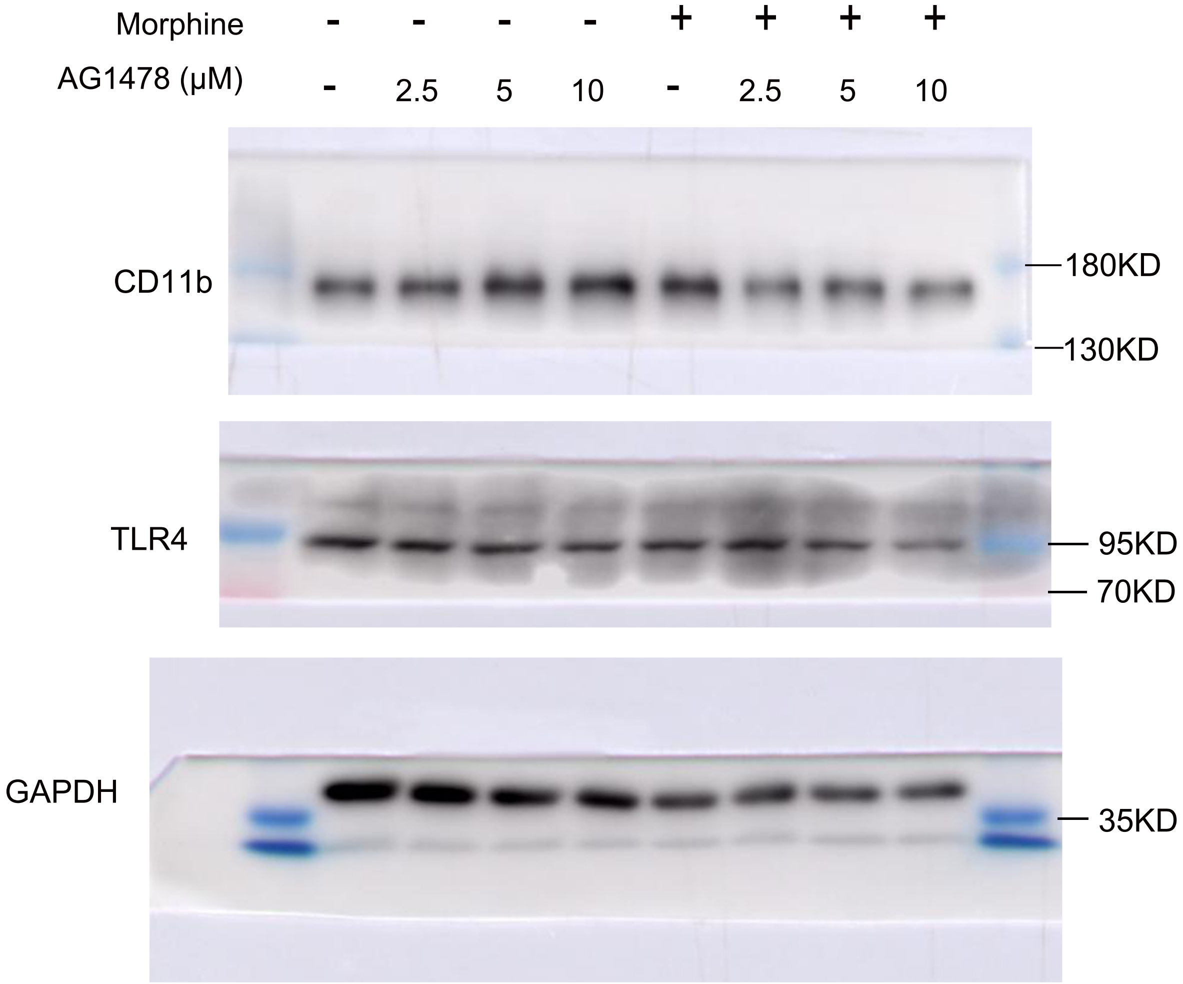
Morphine - - - - + + + +
AG1478 (μM) - 2.5 5 10 - 2.5 5 10
CD11b
180KD
130KD
TLR4
95KD
70KD
GAPDH
35KD

Figure 5b

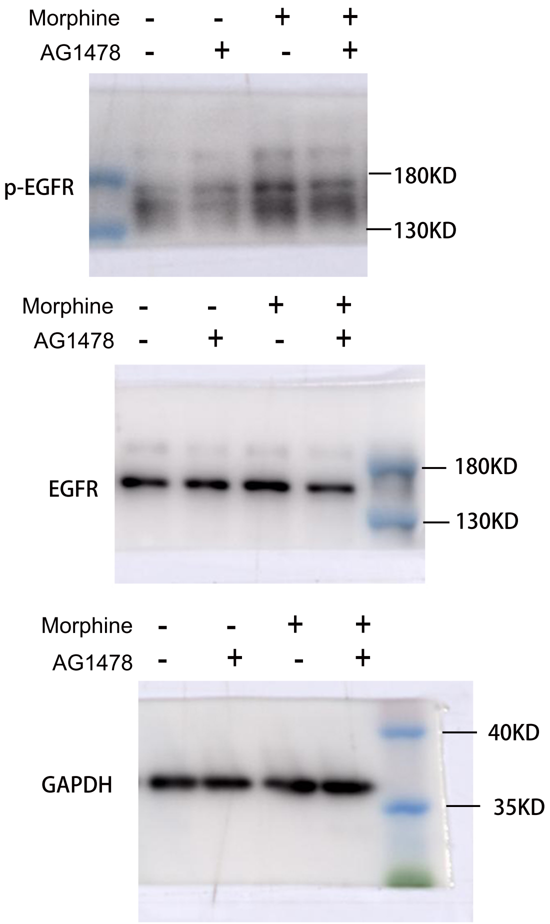

Figure 5c

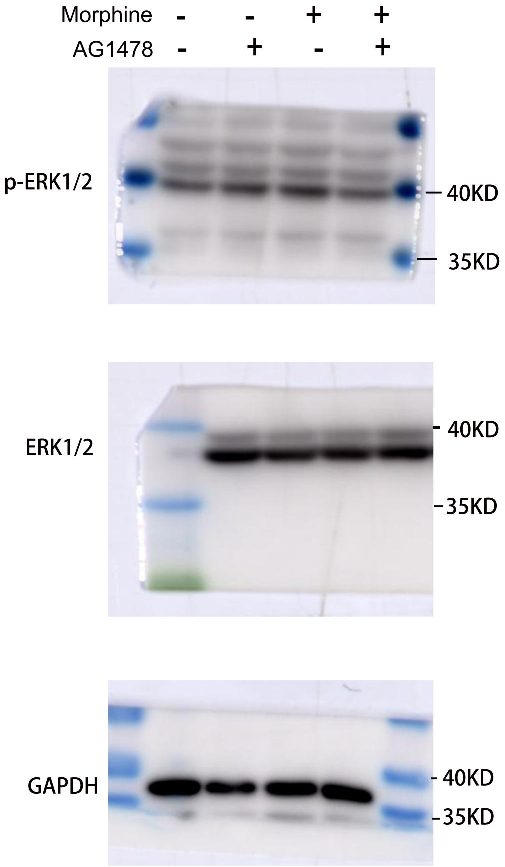

Supplement: S1 Raw images — (PDF) [file pone.0256870.s002.pdf]
